# A New Lease of Life

**Published:** 1889-06-15

**Authors:** 


### A New Lease of Life.

### A Family Complaint.

There was consumption in the family. The doctor elicited that fact when, at Mrs. Curzon's request, he examined her parlour-maid, Helen West. It appeared that the girl's father had died of it, died while he was waiting for admission to a hospital. Twenty years ago hospitals for consumption were few, and the beds they contained utterly insufficient to the demands made on their accommodation. John West had been so far fortunate that he received a subscriber's recommendation for a consumption hospital; and if he had been admitted as soon as his condition was discovered, he might have had many years of useful life. But the beds were full; others besides himself were waiting for a vacancy; and while he waited, the disease developed rapidly, aided by the insanitary conditions of an East-End garret home and the fogs of a London winter. He was nominated for admission in the autumn; he had to wait three months before a bed was vacant; and so the disease gained a complete hold on him, and he entered the hospital only to die within its walls

### Weary of Waiting.

His case was one of thousands. One after another sufferer died, waiting for the help that was not given till it was too late. Meanwhile, subscribers grew angry when they learned that those they recommended could not be received for weeks and months, and threatened to cease their subscriptions if Henry Brown or Jane Smith were not admitted at once.

"As if that would make matters better!" exclaimed the secretary, wearily, as he threw down a letter. "I don't know what to do. Here is Mr. Annesley saying that he won't give us another penny, because a *protégé* of his died before there was a bed vacant for him. I can't help it, I can't make beds; and, apparently, he doesn't see that withdrawing his subscription only makes the chance of some other poor beggar who is waiting his turn worse than it would otherwise be. But there's no way out of it, so far as I can see."

"Yes, there is," said his assistant, to whom, if to anybody, these remarks were addressed.

The two gentlemen were great friends, for they were united in one common enthusiasm—their devotion to the welfare of the hospital. After a proposed improvement had been discussed by the two, neither could have said assuredly in whose brain it originated. But it was Mr. Thorne who to-day suggested the way out of the difficulty occasioned by the paucity of hospital accommodation.

### The Rendall Bequest.

"Where is it?" asked Mr. Deane, the secretary, hopelessly.

"The Rendall bequest!"

The Rendall bequest was the biggest inheritance that had ever come to the hospital. An old lady, who had shown an interest, but only a slight one, in the institution, had surprised its governors—and her own relatives, too, but the latter were digusted as well—by bequeathing to it every penny she was free to dispose of, amounting in all to £100,000. A hundred thousand pounds means an income of £3,000 a year, and this would mean the saving to the hospital officials of an immense deal of worry. Appeals to the public for help would be less frequent and pressing; there need be less resort to charity concerts and bazaars; and the gaunt shadow of debt would not so closely overhang the institution. Three thousand a year would modify the circumstances that now paralysed its usefulness; but it would not compensate for the fact that there were more patients than there were beds for. Mr. Deane pointed this out to his companion, who answered, "That isn't what I should do with it."

### An Audacious Proposal.

"What would you do, then?"

"I would spend the greater part of the bequest—every penny of it, if necessary—in enlarging the hospital."

Mr. Deane uttered an exclamation of surprise. "And who is to keep up the enlarged hospital? For if we spent the Rendall bequest in building and furnishing a new wing, we should have to keep it empty, unless we could increase our income by six or eight thousand a year."

"I suppose the public would have to see to that."

"But the public mightn't see it. The public is uncommonly slow at seeing that it is its duty to subscribe to charitable institutions. And we haven't the pull some institutions have; we can't frighten it into parting with its money with the threat that, if the disease isn't checked, it may spread from the poor to the rich. Consumption isn't contagious."

### Is Consumption Contagious?

"Isn't it!" said a third voice. One of the physicians to the hospital had entered the room during the conversation, and had overheard the last remark.

"Well, doctor, is it?" asked the secretary.

"It is difficult to say," answered Dr. Marshall; "but my own impression is that consumption is to a certain extent a contagious disease. Of course, it is a hereditary one; but we should not let this fact make us forget the possibility of its being contagious too. Now, there's a family I am attending just now in my private practice. The mother isn't a strong woman, and perhaps it is from her the children get their delicacy; but, so far as I can make out, there is no specific lung disease. One of the daughters was at school, and came home helplessly ill with phthisis. The discipline of the school was severe, I suppose, and there may have been some other cause; at any rate, I saw as soon as I was called in that the case was hopeless. The parents saw that, too, but they didn't see that it was their duty to take any precautions; and it was only by accident that I discovered that a younger sister was sleeping with the sick girl—sleeping in a close, stuffy room, because, of course, the invalid didn't like to have the window open.

### The Value of Air.

"It is difficult to make people understand the value of air in chest diseases," said Mr. Deane.

"Difficult, sir," exclaimed the doctor, who was apt to get excited on this topic; "difficult? it is all but impossible! Warm air if we can get it; dry air if it is by any means possible; but, above all, fresh air and plenty of it."

"Hot air alone is no use; it has been a failure when tried as a treatment," said the secretary.

"Naturally. Still air, even when one can find no other fault with it, has the fault of stillness; it lacks the animating power of that which is warmed and dried in Nature's own laboratory, and comes, fragrant with the breath of flowers and odours of the distant sea, to fan our cheeks and stir our pulses. Hot air may give temporary relief in hopeless cases; but, taken by itself, it has little remedial value. It won't cure consumption."

### Is Consumption Curable?

"But will anything do that? asked Mr. Thorne.

"In the rigid sense of the word there is nothing. If you enquire into it closely, there are very few absolute 'cures' wrought by medicine; the part that has once been injured will always be weak. But we can arrest disease, can prevent further mischief to the organ involved, and so prolong life, while by giving attention to the constitution in general we may greatly improve the health. That is what I am trying to impress on the parents of my patient, who were doing what English people usually do when they hear the word consumption—sitting down in despair, content to let their child die without an effort to save her. They didn't want to make her take exercise; they didn't want to do anything but let her slip out of existence with as little trouble as possible. But I've got the conduct of the case, and I'll pull her through. She'll die of old age, or at least of something else than consumption. If I can't cure it I can arrest its progress, and with God's help I will."

"And yet, speaking generally, consumption is fatal," said Mr. Deane.

### Take it in Time.

"That's because we don't take it in time," said the doctor. "I tell you, Deane, my heart gets sore many a time when I examine the patients we get here; so many of them come in just a little too late. A month or six weeks sooner might have made all the difference, but while they have been waiting for admission the disease has been creeping on its way, and has got such a sure hold that they can't shake it off. I'm not one of those who cry out for a promiscuous increase in the number of our hospitals, especially special hospitals. On the contrary, there are one or two that I think might be dispensed with. But consumption is a national disease, one of the gifts of our humid climate, and it is a national duty to provide for the treatment of those who suffer from it. If we had double the accommodation here that we have, we could use it all, and the result would be more than double the amount of relief and approximate cure we now provide."

### Extending the Field.

"Then I suppose you would back Thorne's advice with regard to the Rendall bequest?" said Mr. Deane; and he told Dr. Marshall what his colleague had suggested.

"That is the best thing I have heard for a long time," exclaimed the doctor. "Deane, you must press it upon the governors. Tell them it's the thing they ought to do; tell them it is salvation to a host of sufferers, and that if they refuse to do it because of the increased responsibility, they are cowards and not Englishmen."

"Well, I won't put it that way," said the secretary, "but I'll do all I can to persuade them."

By this time the enthusiastic doctor had got into a day-dream.

"We need not confine ourselves to consumption then; we could take in all chest diseases, and get them in the early, the inflammatory stages. There's pneumonia—we don't attend to pneumonia sufficiently; and there's the great field of chest surgery almost untouched. I believe that if we had a larger hospital, and could take in a greater variety of cases, we could do untold service, not only to our own patients, but to the cause of medicine in general. I vote with Mr. Thorne for the new hospital; but there's one apartment which it must have, and I must leave it to you to press the necessity of it on the governors."

### An Entertainment Room.

"What is that?"

"An entertainment room."

Mr. Deane shook his head. "I don't know that the governors will approve of it. You know what subscribers are. They don't mean unkindly, but they are so apt to think that anything in the way of extra comfort or pleasure is waste of money."

"I wish I had some of them in a hospital ward," said the doctor, savagely. "I'd keep them for a fortnight in the way they advise our patients should be kept. They should have as much wholesome food as they needed, but no variety to tempt the appetite. The wards should be as bare as a prison cell, not a flower or picture about, and nothing at all in the shape of amusement for them. They would change their minds before long, I'd wager, and recommend most strenuously that hospitals should be made as bright and cheerful as possible."

### The Worth of Amusement.

"Then, doctor, you should write a book on the medicinal value of amusement," said the secretary, smiling.

"No, I won't. I'll write about anything that can be proved by material means—by scalpel and microscope; but there's no use addressing to the public anything that appeals to the imagination; above all, I won't pretend to explain anything I don't understand myself. Now I understand how the existence of diseased lung-tissue interferes with breathing, and I can make other people understand it. But I don't know how it comes that if we get up a concert in one of our wards the patients are better next day; I only know that it is so. But since I am sure of that fact, I will plead with all my power for the means of making these entertainments better and more frequent; and I know that in doing so I am pleading for the health of our patients. I might argue the matter for ever, and my arguments be all wrong; but I am sure of the fact, and I'll stick to that. And I think you'll both bear me out in it."

"Yes, we will," said the secretary, "and I'll do my best to get the entertainment room you want."

### How to Get In.

It took some time to bring the governors to unanimous approval of the new hospital, and all the improvements that were suggested for it; but the party of progress carried the day, and the new building arose. It was to it that Helen West was taken. There had been a little difficulty about her admission, for neither her mistress nor anyone she knew was a subscriber to its funds, and a letter of recommendation was needed to procure her entrance. At last, however, among Mrs. Curzon's friends was found a lady who had subscribed for years to the Hospital for Consumption, and she at once promised the necessary recommendation.

"I am so much obliged to you, Miss Ashton," said Mrs. Curzon. "West is such a nice girl, and if the hospital treatment will do her good it would have been a shame if she couldn't have got in, just because she happened not to know anyone who subscribed to it. You know, *I* think," she added with emphasis, "that a case ought to be admitted on its merits, without the need of bringing influence to bear."

"I quite agree with you," said Miss Ashton. "Nothing is more sad than to see a suitable case passed over because preference must be given to one recommended by a subscriber; but what are the officials to do? In the present imperfect condition of human nature so many people won't give their money unless they get some privilege in return, and the money must be got, or how are the patients to be kept?"

### Is the Money Wasted?

"Don't you think the officials may waste it?" asked Mrs. Curzon, more in jest than earnest.

"Not wilfully. Of course it would be rash to say of a hospital, any more than of any other establishment, that there is no extravagance in its management; and I know that sometimes large sums are spent uselessly. For example, there was a great deal expended some time ago on an elaborate hot-air apparatus. It was to do wonders for the cure of consumption, and it failed almost completely."

### Try All Things.

"What a shame! They ought not to throw away the money entrusted to them in that way."

"Don't say that. In trying to save human lives we are bound to use every means, for that is the only way in which we can learn what is best. I'm sure I don't grudge my money for such purposes."

"Well, I should grudge mine," remarked Mrs. Curzon.

"As you don't give any, it doesn't matter much," said Miss Ashton, rather sharply, for it had struck her several times in the course of the conversation that Mrs. Curzon, the wife of a rich shipowner, might at least buy the right to criticise the hospital as freely as she did. "People always give opinions when they won't give anything else."

Mrs. Curzon blushed a little. "One has so many expenses," she said, rather uneasily; "and somehow I had never had anything to do with hospitals till poor West fell ill and I wanted to get her in."

### A Reason for Preference.

"Well, now that you have been brought more or less into contact with them and their work, I hope you will show a practical appreciation of it."

"But there are so many calls on one's purse. One can't give to everything, and, really, there seems no reason for preferring one institution to another."

"One can always find a reason for choosing one form of charity rather than another. And if I am not mistaken there is a reason, Mrs. Curzon, why you should take a special interest in a consumption hospital. Were you not threatened with consumption yourself when you were a girl? I think I have heard you say so."

"Why, yes, I was. My sister died of it, though no one had ever suspected its being in the family. Both my parents are alive now, and well and strong, considering their age. But poor Lucy died, and it looked as if I was going to follow her, when dear old Dr. Marshall pulled me round. He certainly saved my life."

"Well, he was one of the physicians at the very hospital to which your maid has now been sent. It was there he studied the treatmen which, you say, saved your life, and there his memory is honoured to this day."

"Really? Oh, then, I should like at least to see the hospital; and I mean to visit poor West while she is there. Would you go with me some day?"

"With pleasure; but I should like to make one condition."

"What is that?"

"That Mr. Curzon goes too."

"Why do you want him?"

"Can't you guess? Because a man's cheque-book is worth more than a lady's purse."

### Seeing for Oneself.

Accordingly, they went a few days after Helen West had been admitted. Mr. Curzon was in attendance, like a dutiful husband, though he laughingly told Miss Ashton that he had left his cheque-book at home;

"Or rather, to make things quite sure, I locked it up in my desk in the City."

"Perhaps you'll regret that before the day is done but it really doesn't matter much," she answered. "I don't mind making a journey in a good cause. I can easily call on you in the City to-morrow."

Miss Ashton was determined that her friends should see the hospital thoroughly; so she had sent a note to the secretary warning him of their coming, and asking him to show them all he could. As she was known to be a loyal supporter of the hospital her request was readily complied with.

### Free-will or Force?

"Why, I've passed it ever so often," exclaimed Mrs. Curzon, as they came in sight of the red brick building, with the neat terra-cotta mouldings round the doors and windows.

"Well, you could hardly help knowing what it was if you passed it," said Miss Ashton, smiling, as she pointed to a large board, with the inscription, "Hospital for Consumption, supported by voluntary contributions."

"I wonder you never went in," she added.

"I couldn't be bothered. And, you know, there are so many hospitals. 'Supported by voluntary contributions' stares one in the face all over London."

"I sometimes wonder if it will do so much longer," Miss Ashton remarked thoughtfully. "Sometimes I think it will have to be painted out, and 'supported by a tax upon the nation' substituted for it. Unless the friends of hospitals put their shoulders to the wheel, unless rich and poor combine to support them, aid will have to be sought from the State. At present, it seems as if our population would outrun our charity. Not merely in London, but from all over the country comes the cry for help. In some places there are large districts—whole counties even—with no hospital accommodation whatsoever; in others the accommodation is there, but for lack of funds beds have to be kept empty. And everywhere institutions are in debt."

### How the Wrong People get in.

"It's bad business, you know, Miss Ashton, living beyond your income," remarked Mr. Curzon; "though I have heard that hospitals are never flourishing unless they have a good big debt. For my part, I think that if all the people who shouldn't be in hospital were turned out, there would be plenty of room for the remainder, and plenty of money to keep them, too."

"There is truth in what you say—a half-truth, about which Tennyson says some nasty things that I won't quote to you. But as a matter of fact, if you would only give the hospitals more money, the doctors would be able to do just what you want, and select their patients simply because they were suitable, and without fear or favour of the subscribers."

"That's all very well, Miss Ashton, but as a matter of fact on my side, I'm not going to give my money to anybody to do as they like with. I like to keep an eye on it, and see that it is spent properly."

"I am so delighted to hear you say so, Mr. Curzon," exclaimed Miss Ashton, with a rather mischievous look.

"May I ask why?"

"Because you evidently mean to give enough to the hospital to qualify you for the post of governor; and what is better, you mean to take a personal interest in its management."

"I assure you, I——."

"Here we are," said Miss Ashton, interrupting him a s they reached the porter's lodge; and the intended protest against rash assumptions was never finished.

The Compressed-Air Chamber.

"And what shall I show you first?" asked the secretary, when they had reached his office, and Miss Ashton had introduced her friends.

"I think it would be best to begin with the engine-room and the basement generally."

"We might take that for granted," said Mr. Curzon. "Engine-rooms are pretty much alike all the world over."

"Perhaps they are," the secretary admitted. "We have, however, one thing that you can't have seen a great deal of, for there are only two specimens in the world, one in Paris and one here. I mean the compressed-air chamber."

He led the way to the chamber he spoke of. It was a small round room, or rather a hollow pillar, made entirely of strong iron. It was illuminated by port-holes of thick glass, like those of a ship, and was closed by an iron door, studded and barred to increase its strength. Inside were two or three chairs.

"What a gloomy place! It looks like a condemned cell," exclaimed Mrs. Curzon. "It would make me feel ill to enter it."

"If you had to enter it you would probably be too ill to care about the look of it, and, having once been in, you would appreciate the relief you got in it so much that you would never dread it again," said the secretary. Then, turning to Mr. Curzon, he began to explain the use of the chamber:

"When our patients get so weak that they cannot inflate their lungs we bring them in here for a couple of hours, and pump in air to them. We can bring a pressure equal to ten atmospheres on to them—that is, 10lb. to the inch, or 700lb. on the whole body. They find the dense air easier to breathe than the ordinary. You know, perhaps, the feeling of gasping that comes from breathing too thin air; this dense air has just the opposite effect."

"I should think it would choke them."

What it Does.

"On the contrary, it relieves them immensely when they are in the condition I speak of, and we find it especially useful in cases of asthma. Of course, we are careful that the air the patients get is good. It is drawn through this cylinder, which has several layers of cotton wool in it to absorb dampness and impurity. Sometimes, if they are weak, they feel a little faint at first; but there is always a nurse in the chamber with them, and she can signal to the attendant outside, who then takes the pressure off this double window with the little passage between the two frames, and passes in the brandy, or beef-tea, or whatever is needed."

"But doesn't your compressed air escape meanwhile?"

"No, for only one side can be opened at a time. Not until the outer pane is closed can the inner one be opened."

In the Hospital Dispensary.

They were taken to see the Turkish bath, and afterwards conducted to the out-patient department, where, in different rooms, men and women were waiting to see the doctor, or have their medicine renewed, going on to the dispensary to have their prescriptions made up. The dispensary had the aspect of a large, busy, and well-provided drug-shop. Lozenges of every kind filled the drawers; there were powders done up in papers of different colours, so that the chance of one being mistaken for another was reduced to a minimum, and besides the numerous bottles of different medicines, there were great flagons of cod-liver oil, with taps to draw it off by.

"I suppose you use a good deal of oil in a year," said Mr. Curzon.

"Between twelve and fourteen hundred gallons," was the reply. "Then we buy about a ton and a half of lozenges—33cwt., to be accurate—and about 2cwt. of pills, not counting what we make."

"How on earth do you get through that quantity of physic? And people say that drugging is going out of fashion."

"We can't do without drugs altogether yet, and with as many out-patients as we have, a good deal of medicine disappears every twenty-four hours. Remember, we often have as many as four hundred out-patients in a day."

Refreshments.

"But if you have so many, some of them must wait for a long time before their turn comes, and that is so tiresome, especially if they come from a distance," said Mr. Curzon.

"In that case they can get refreshments here," said the secretary, leading the way into a corridor, where a buxom woman was selling tea, coffee, lemonade, and buns. "Our porter's wife sells them anything they want at a cheap rate, which is often a great boon to them."

"She does it as a private venture, then?" asked Mr. Curzon.

"Yes."

"I don't like that. It is a blunder, believe me—I am speaking as a business man—to let anyone belonging to the establishment make a private profit out of it. Perquisites usually are a blunder. The hospital should do it."

"The fact is that the profit would be too small for it to be worth our while to take it up as part of the institution, and, of course, the patients don't care who provides the refreshments so long as they are good and cheap. Still, I see the force of your contention."

Obstinate Patients.

Leaving the out-patient rooms the party glanced at a covered court where a number of men were playing at quoits, and enjoying with about equal zest the sunshine and their pipes.

"They like this court," said the secretary, "because they can smoke here. There's no doubt that many of them miss their tobacco; but, of course, we can't allow them to smoke in the wards. It's strange, though, that some of them fail to see that this is a reasonable restriction. There was one man actually went out o the hospital last week because we could not permit him to smoke in bed. I felt very angry; we could have done him so much good, and he wouldn't give himself a chance."

"Do you have many patients go off in that fashion?" asked Miss Ashton.

"Not many; there are very few so self-willed as that stupid fellow. If we can get a patient to stay ten days in the hospital he is pretty safe to stay out the time he is admitted for; but some are homesick and insist on going away after a few days. We have one who is determined to leave us and go back to Peterhead—Peterhead of all the bleak places in the world!—just because he is lonely. He admits that we have done all we could for him here; he even admits that he is better since he came in, but he is homesick, and he is determined to go."

Patients from all Quarters.

"Peterhead," said Mr. Curzon. "That's in Aberdeenshire, is it not? Do you take patients from there?"

"We take patients from every quarter of the kingdom," said the secretary. "I could not at this moment give you an accurate list of all the patients we took in last week; but I know that one came from Cornwall, one from Huntingdon, and one from Norfolk. We get quite as many from the country as from London; and this is true of all consumption hospitals. I am sure that Ventnor could tell the same story, and the homes at Bournemouth and other places, too."

"And does the country contribute much to your funds?"

"Almost nothing. A special hospital is much more likely than a general one to get patients from all quarters just because of its special character. But it is only very rarely, unless indeed some well-to-do person is interested in the patient, that any money comes from the same quarter."

"But can't you do anything?"

"We mention the fact in our annual report, but I don't know that much comes of it. It is not that people are mean, but that they don't think of the matter."

Pianos and Pictures.

Before going upstairs they looked into the entertainment room, which Dr. Marshall had struggled so determinedly for long ago. Its value was proved and undoubted by this time. But to Mr. and Mrs. Curzon, who had never seen anything of the kind before, it seemed a useless luxury. It was certainly a very pretty room to form part of a charitable institution. At one end was a miniature stage, on which stood a grand piano. On either side were rooms for the performers; the curtain and footlights were all *de règle*. But the walls were covered with oil paintings, which Mr. Curzon, who was something of a connoisseur, pronounced to be of considerable value. While the ladies were trying the piano, which was a very good one, he was studying the pictures.

"I don't approve of it," he burst out at last. "I don't want to be uncivil; but I think this is all sheer waste. What do the people who come here want with pianos and pictures? Subscriptions aren't given to be spent in that fashion."

"No subscriptions are spent in that fashion," said the secretary. "The piano was a gift. I am thankful to say that we have pianos in almost every gallery. Many of our nurses, and occasionally some of our patients, can play, and the others enjoy listening immensely. By-the-bye, Mrs. Curzon," he added, turning to that lady, "we store pianos, too. We have one just now that the owner asked us to keep for him while he was abroad. You see, he knew that with us his instrument would be kept in a place with a suitable temperature, and would neither suffer from not being used nor be roughly handled. We should be delighted to keep yours for you while you are away on your summer holiday."

Treasures in Canvas.

"But the pictures!" reiterated Mr. Curzon. "I can see they are of value; how did you get them?"

"They were left to us by the old lady whose bequest of £100,000 enabled us to enlarge the hospital. We realised almost everything else, but kept the pictures."

"Hadn't you better sell them, and spend the money on your sick people?"

The secretary smiled. "I don't wish to institute discourteous comparisons," he said, "but doubtless you remember who it was that was of opinion that a certain box of ointment had better have been sold and the two hundred pence given to the poor, than expended on the Giver of health. And I think that our pictures do good where they are. Healthy people don't, can't realise the worth of a pleasant sight or sound to an invalid. The patients like coming to this pretty room to hear a concert or see some little play. It does them good, as the doctors will tell you; and if you will only come to one of our entertainments next winter you won't doubt how much they enjoy it. I am sorry that we have just stopped them for the season. In summer, with the sunshine and the long days, we don't need them so much; but they are of great value in the winter."

"Possibly," said Mr. Curzon, but he did not seem to be convinced.

The Patients' Verdict.

The secretary said nothing, but as they were going upstairs to see the wards he paused before three women who were sitting on a sunny window-ledge on one of the buildings. He asked one of them—a pretty girl with bright eyes and a hectic glow on her cheeks that seemed all the more vivid against the transparent pallor of her skin—a few questions about her health. Then he said:

"You have been in some time, I think. You came before the entertainments stopped."

"Yes, sir; just before the last one. I wasn't feeling well then, and Sister Victoria wasn't sure that I ought to go; but the house surgeon said it would do me good."

"And did it? I forget what we had that last entertainment."

"Do you, sir?" she exclaimed incredulously. *She* had not forgotten the details. "There was a concert first—oh! you must remember the young lady that played the banjo; she was so pretty, and had on such a lovely dress. She sang 'Wait till the clouds roll by,' and asked us all to join in the chorus. And we did, with all our hearts. And then there was that funny play 'Bear and Forbear.' I don't know when I laughed so much."

That Banjo.

"But didn't it tire you?" asked Mrs. Curzon, who could not think of an invalid caring for a banjo solo.

"Oh! we were tired at the end of it, ma'am; but then we slept the sounder for it, and I at least woke up next morning brighter than I had felt for many a long day.

And now, when I feel a bit tired, I always sing to myself 'Wait till the clouds roll by.'"

"That's a pretty good account of our entertainments, I think," said the secretary.

"Yes," said Mrs. Curzon. "But I could'nt help thinking that was a funny song for a young lady to sing."

"Ah! the lady in question has often come and helped us, and chooses her songs to please the patients, rather than to show off her own talents. I won't tell you her name, but it is very well known in society. And she always pays us the compliment of coming in evening dress, which is greatly appreciated by the audience, especially the female portion of it. It gives them something to talk about for days after."

"Who is the Sister Victoria the girl talked of?" asked Mrs. Curzon. "Is that her real name?"

"No; it is her title. She is sister of the Victoria gallery. We go on the principle that the queen never dies; and though sisters come and go, through all changes we have a 'Sister Victoria.' Here we are at the door of the gallery; now we shall see her."

### In the Gallery.

He opened the door, and ushered the party into a long wide gallery, very lofty, and lighted by numerous large windows. Comfortable seats and couches lined the walls, and in the centre ran a cross gallery of smaller size, the greater part of which was occupied by a dining-table, now—between meal times—covered with a crimson cloth. At the other end this gallery branched out into a large bow-window, about the size of a small room, which was occupied by a piano and a number of tables, all plentifully supplied with flowers and growing plants, many of them in handsome china pots. There were bookshelves against the wall, and, if one could judge by appearances, the books they contained were in frequent demand. About the gallery were dotted little groups of women, reading, sewing, and conversing. One or two of the feebler ones were lying on the comfortable cane couches brightened with red cushions, but the most were seated on the chairs and benches. They were of all ages; some were more cheerful than others, but none looked discontented. On one side of the gallery, doors gave entrance to the various wards and to the ward kitchen, where preparations were now going on for the patients' tea.

One or two nurses were moving about, and Sister Victoria came out of her little room at the end of the gallery to meet the party. She was a tall, pleasant-faced woman of middle age, with a keen but kindly expression. One could see as she came down the gallery that the observation of every detail of her surroundings was an instinct with her, and that she was more than a nominal ruler in her domain. She would not be harsh, but she would expect obedience. But it was evident that while the patients respected her, they did not hold her in any fear. For one old woman stopped her as she walked down the gallery to ask her opinion about the shape of a cap she was making for one of the nurses. Sister Victoria stopped and listened to the explanation of the needlewoman's difficulties, deftly showed her how to stitch it into the required shape, and spoke a word or two of praise about her sewing before she passed on to greet the visitors.

"Your patients all seem happy and busy, sister," said the secretary.

### Woman's Privilege.

"Yes; they are happy because they are busy," said the sister. "That's one advantage the women have over the men; they can always do a little work; they don't feel as helpless and useless as if they were quite idle. Men get tired of reading sooner than women get tired of sewing or knitting, and draughts and dominoes pall upon them soon."

"Why don't you teach the men to knit?" asked Miss Ashton. "I can never understand why anything like that should be considered unmanly."

"Ah! Miss Ashton, you are one of the advanced ladies, who want the women to go out into the world while the men stop at home," said Mr. Curzon.

"Not I! I think in a general way that our present division of labour is a good one. Women's sphere is home, and all the work that is done there belongs to her. But when, through illness, a man is compelled to remain at home, why should it be beneath his dignity to take up any work that would keep him from feeling dull and wearing out his wife's patience?"

### In the Wards.

Sister Victoria led the party into the wards, in which were the severer cases, where the patients were not able to get up. Naturally, there was an element of sadness here that was not to be seen in the galleries; but few of the patients uttered a complaint. They were "not so well to-day," that was all. The wards were all small, which gave them a home-like look they would not otherwise have had. None contained more than eight beds, many only four, and some two. The inmates of each ward formed, as it were, a little family by themselves; they felt a closer union with each other, even when they mixed with the larger household in the gallery. But the change from ward to gallery, the possibility of spending the day in an apartment where they did not spend the night, lessened the monotony of hospital life, and gave it a more social aspect—a thing not to be neglected in dealing with consumptives. The walls were brightened by a few cheerful illuminated texts, and in all where there were any occupants—in some all the patients were up—was a vase of flowers.

Mrs. Curzon noticed, too, that in some—not all—there were painted tiles round the fireplace.

"Those," said the secretary, in answer to her question about one particular set, "were done by the wife of one of our physicians. We never spend money on such luxuries; but, of course, we are very glad to get them when they are given to us. We have a decoration of some sort round the fireplace in most of the women's wards—now *place aux dames*, you know. We are waiting for a few more kind friends to smarten up the men's abodes."

### Memorial Beds.

"I see there are names above the doors of some of the wards, and plates with names above many of the beds," said Mrs. Curzon.

"Those are memorial wards and beds. They are mostly records of great sorrows. You see there are plates of the same shape above all the six beds in this ward, but only four have names on them. They are the gift of two ladies, the survivors of their family. They first named two beds in memory of their parents, then after a while a third, and a fourth in memory of two brothers. Two other brothers have died, and they have asked us to keep these two beds for them 'till they can afford to name them also as a memorial.'"

"Then 'naming' a bed, as you call it, is an expensive business," said Mr. Curzon.

"It costs at least a hundred guineas. And, of course, to name a ward is more costly still. But we find people who have lost those they love ready to pay for such a memorial. And it is better than setting up a costly gravestone. It does good to the living as well as honour to the dead. We have now five memorial wards and twelve memorial beds, and we hope to have more."

### A Touch of Chivalry.

Climbing another flight of stairs, the party entered a gallery which, with the wards attached to it, was devoted to men. There was undeniably more *ennui* here than in the women's wards; more of the patients were avowedly idle, and of those who were reading and playing draughts not all seemed much interested in their occupation. Hospital life is undeniably more trying to men than to women, who are more accustomed to indoor work, and can carry on some part of it in the institution. On the other hand, nurses say that male convalescents are more ready than female ones to render those little services in attending to their fellows and carrying things for the nurses which patients can so easily do.

"I don't know," a nurse said once, "whether it's a touch of chivalry in the men that makes them anxious to help us, or if the women are determinedly idle only because they are so tired. Poor things! they have such a hard life, such endless drudgery at home, that many of them have the same ideal of hospital comfort that the charwoman had of heaven—they want 'to do nothing for ever and ever.'"

### A Patient of another Class.

The appearance of the visitors formed a welcome break in the monotony. All looked up except one man, who was busy with a letter. He was writing home. This gallery was not quite the same in shape as the other. The patients had become too numerous for the dining-hall in the centre, so a corner wall had been broken down, and the gallery enlarged into a room, mostly of glass, which resembled a conservatory, and filled with plants.

Looking into one of the wards, Miss Ashton noticed a young man whose delicate features and general aspect suggested that he was of a different class from most of the patients. He turned away his head as the visitors looked in, and was evidently anxious to avoid observation, but Miss Ashton recognised him.

"Please go on," she said to the others. "I see a friend here; I'll join you presently."

She went forward to the young man, who was seated on a chair by the window.

"I am sure I know you," she said. "You are Dr. Evans, who used to be in Princess-gardens, are you not?"

"Yes," he answered, with a deep flush and a look of vexation. "I did not think anyone would have recognised me here."

"Ah, you reckoned without an old maid! We have all prying eyes, you know; and I happened to live quite near you, and saw you pass every day. I noticed in the winter that you looked ill. No wonder; we had terrible weather, and a doctor's life is so hard."

"I don't think it was my professional work that hurt me," he said bitterly. "There wasn't enough of it."

"I wish you would tell me all about it," said Miss Ashton. "I'm old enough to be your mother, you know"—which was rather stretching a point, in view of her forty years and the patient's thirty, but she was determined to win his confidence—"and I have a reason for being interested in young doctors."

### A Sad Story.

"It is nothing very new or strange," he answered. "I was poor, but I was determined to be a doctor. I overworked myself, and probably was not too kind to myself in other ways during my student career. I thought that when I had gained my qualification I should need nothing more. Well, I was wrong. I thought it was possible to make a practice in London by dint of brains and energy, in spite of the lack of money or influence. I did not know what London was."

"'The terrible city, whose neglect is death,'" murmured Miss Ashton under her breath. "I know what the struggle is. One man in a hundred, with a strong will and an iron constitution to back his abilities, may conquer in it, but for the rest——"

"For the rest the end is defeat and death," he said, ending the sentence for her.

"I hope it won't end that way for you," she exclaimed, fervently. "You are better now, are you not?"

"Much better; and I can't speak too gratefully of the kindness and consideration I have had here from everyone. I am to go to a convalescent home soon. After I leave there——"

"Well?"

"I shall try to get a post as assistant somewhere in the South, and so struggle on till the end comes."

"Don't think of the future too despondently," said Miss Ashton. "Consumption is something like heart disease; one may have it all one's life and die of something else at a good old age. It depends a good deal on taking the disease in time, and being careful of oneself afterwards. May I come to see you again, now that I have found you out?"

"Certainly," said the young doctor, looking brighter than he had done. "I have rather avoided the ladies who visit us occasionally; but you are different."

"I see; you are afraid of being patronised and 'poor thinged,' though I really don't think any of us who visit the hospital are very guilty of that. But I shall certainly come back to see you."

### The Separate System.

There were tears in Miss Ashton's eyes when she rejoined her friends and told them the story she had heard.

"I am so glad you have gained his confidence," said the secretary. "We occasionally get patients like him, to whom the bread of charity is bitter exceedingly, and they are difficult to deal with. I don't mean that they are troublesome in any way; on the contrary, one may note in hospital patients that the more accustomed they have been to refined surroundings and the courtesies of life, the more amenable they are to discipline. But one cannot help knowing that to depend on others is galling to them, and one is afraid of seeming to play the patron in every word one utters."

"I can understand that," said Miss Ashton. "Would not a case like this be very appropriate for treatment under the separate system? I mean as in that hospital where every patient has a separate room."

"Yes; that system is an excellent one where it is practicable, and the hospital you speak of has the advantage of being situated in an admirable climate for consumptives. But it is expensive. Patients have to pay a weekly sum, and though there is nothing to be said against this when they can afford it, it renders the hospital out of the question for a large number of cases otherwise appropriate. And, after all, I am not sure that the results there are better than in other hospitals."

Give Him a Chance.

"I want you to do me a favour," Miss Ashton went on, turning to Mr. Curzon. "Some of your vessels go to Madeira, don't they?"

"Yes."

"And you have correspondents there—men of some influence, I suppose?"

"Yes, I have."

"Then will you give this poor delicate young doctor a passage out, and a letter of introduction to anyone you know who might help him? It is quite possible, you know, that in a better climate he might live a long time yet. It is so sad to think of a young man having to lie down and die in the prime of life before his work is done. You will do what I ask, won't you?"

"Oh, certainly. It won't cost me much trouble, and it's worth trying if it gives the poor fellow another chance."

In the Kitchen.

"Now," said the secretary, as they began to ascend another flight of stairs, "I'm going to show you our kitchen."

"I want to see that," said Mrs. Curzon. "In fact, I wondered that you didn't show it us at once, when we were on the basement, and we are still going upstairs."

"Still we'll come to it presently," he answered, smiling.

And so they did, at the top of the building instead of at the bottom, as in most houses.

"How awfully funny!" exclaimed Mrs. Curzon. "Does it answer well? It doesn't seem right, you know."

"It answers admirably. Now perhaps, at home, you are occasionally worried by the smell of dinner before the dinner appears?"

"Often; it's so horrid."

"You see, it's in the nature of smells to rise and not to fall. Now, if our soup or mutton does betray its presence in process of cooking, the smell floats upward into the free air of heaven, and doesn't trouble our patients, whose appetites, fastidious through illness, might be spoiled by it."

"I understand. But don't your tradesmen grumble at having to climb up here for orders, and to leave things?"

"That, of course, is a reason why the attic kitchen can't conveniently be adopted in private houses; but it doesn't affect us here. The orders for the tradespeople are given at the steward's office on the ground floor. A list of the food required for each ward—the diet, as the patients' allowance is called—is sent daily, and from it the number of legs of mutton, the amount of fish, vegetables, milk, and the rest are calculated. The food itself is sent up by the lift, so that everything works smoothly."

Gas or Coal.

"Now, I want you to admire our methodical ways," he went on. "I fancy, you know, that we are much more careful in hospitals that most people are in private life. A careful housekeeper has a fish-kettle and a milk-saucepan, but I don't think she goes much further in the direction of keeping each vessel for its own purpose. Now, look at our saucepans. They are cauldrons, as you see, and each is supplied by its own gas fire. One is kept for milk, one for beef-tea, one for soup, one for boiling meat. There's no danger of the flavours getting mixed."

"And I suppose there's one for potatoes?"

"The boiling of potatoes is a relic of barbarism, my dear madam. We steam ours in this cupboard."

He opened a tall iron cupboard or oven with a number of shelves in it, and showed the party how the steam was supplied. Then he showed them the great roasting oven. The neatness and cleanliness of the whole charmed Mrs. Curzon.

"You really must get me a gas-stove, John," she exclaimed; "it would be so much handier. The heat is there whenever you want it, and you can turn it off when you don't. Then it is steady and constant. There's no excuse for cook sending up a joint half-cooked because the fire wouldn't burn, or a pie burnt to a cinder because the oven is too quick. It is ever so much nicer than coal."

"And ever so much dearer," said Mr. Curzon.

"Well, we don't find it so," said the secretary. "The extra cost is made up by the economy in servants. Now we have two kitchens in this hospital, because, the building being divided into two parts through the exigencies of our site, it was impossible to send the food hot all over the place. When we built the new portion of the hospital we had to give it a kitchen of its own. In this the food for 137 patients is cooked by one cook and a kitchen-maid. In the other are prepared the meals of the remaining 184 patients, and also of the resident medical officer and other officials—that means a late dinner as well as a midday one—and there the cook has two kitchen-maids to help her. That doesn't seem great extravagance, does it? And, of course, if our arrangements were not so carefully made to produce the minimum of trouble, we should want a larger staff of servants, whose food and wages would soon more than balance the initial cost of our machinery and our expenditure."

Clouds and Their Linings.

"I need hardly take you through all our wards," said the secretary; "one is pretty much like another. In each you will find a good deal to sadden you; in each you will find material for the comforting thought that though disease cannot be avoided it can be mitigated. Temporary relief is better than nothing, and in many cases the relief is more than temporary."

"But," said Mrs. Curzon, "I want to see my servant, who is in the hospital."

"I know, and I shall take you to her now, but as Miss Ashton told me you had not seen our hospital before, I wanted to give you a general idea of the place the girl was in. So many people have such an idea that it is 'all hope abandon ye who enter here' with those who come to us, that I wanted you to know that our cloudy life has a silver lining. With regard to the patient you are interested in, I am happy to say that the doctor's report of her is very cheering. She has improved greatly under treatment, and we hope that before very long she will be fairly well again."

Helen West.

In a few minutes they were face to face with the girl. She was not a very sturdy-looking creature, but there was no special look of illness about her. She flushed with pleasure when she saw her master and mistress, and while the other members of the party strolled along the gallery chatting to the other patients, Mrs. Curzon sat down by Helen West's side to hear all she wanted to say about her life in hospital.

"Everybody is kind, ma'am. It's not only that they do you good, but they're so kind and pleasant with it.

The nurses make you feel that it's a pleasure for them to do anything for you; and the patients that have been in a bit longer than you are so anxious to make you feel at home—for just at first, you know, ma'am, one does feel a bit lonely. And then —— Oh! Mrs. Curzon, ma'am, what do you think of a princess coming to play the piano to the likes of us? You know somehow it makes one think more of oneself—it makes one more determined to live and get well again, when you feel that a lady like her cares about you. I think everyone of us thought a deal more of ourselves after the princess had been here."

"I daresay you did, West. And I am sure if the princess knows it, it is the best reward she could have for the trouble of coming. I hear that you are getting on very nicely. You must get well fast, for I am keeping your place open for you; and I'll try to make it an easy one.

### A Samaritan Fund.

"Thank you, ma'am; it's very good of you to do that. I'm so sorry, sometimes, for the patients here when they go out. They're better, but they're not fit for hard work, and some of them have neither home nor friends. But I heard the sister speak of a fund that helps them a bit. It is used to pay their railway journey if they come from the country, or buy them a bit of warm clothing if they need that, or give them a fresh start in the world in some way. That must be a great comfort when they have nobody else to help them."

"I'm sure it is. When one comes to think of it, what a lot of help can be given in this world. But somehow one doesn't think. Well, when do you think you will be able to go out?"

"I don't quite know. The doctor could say better than me. But, if you please, ma'am, even if I am out, would you mind my coming back to the hospital to be confirmed?"

"To be what?"

"Confirmed. I never was, you know; and the chaplain here has a class, and is preparing a good many of us—both out and in-patients. And somehow I feel as if, supposing I get better, I would like to make a fresh start in every way from the hospital."

Mrs. Curzon readily granted the desired permission; but when she had rejoined the secretary she said, "I did not know you gave religious instruction in the hospital."

### The Question of Creed.

"It is not compulsory," he answered. "We would be very loth to meddle with anyone's faith; but, as you may guess, there are a good many that come here who simply have never thought of religion at all till illness brought them leisure. Catholics and Nonconformists can have their own priests and ministers, and I am happy to say that we are on the best of terms with the clergy of all denominations in the neighbourhood, and anyone of them will come to visit those of our patients who share their form of belief without trying in any way to influence others. To those who belong to the Church of England, and to the many who have no creed at all, our own chaplain ministers, and we have had many most touching confirmations and communions in our chapel. I must show you the chapel. It is a very pretty little building, and is duly consecrated. The patients speak often of the satisfaction they feel at having a proper chapel to worship in instead of a mere empty ward, as there is in so many hospitals."

### An Object Lesson.

While the secretary was speaking, the door opened, and an elderly woman, with a rosy rustic face, came rather shyly into the ward.

"Do you want to see anyone here?" asked the secretary.

"No, sir," she answered, "but I just thought I would like to look into the hospital and see my old ward, and the porter said he thought I might."

"You are an old patient, then?"

"Yes; me and my husband came up from Leicestershire to-day to go to that exhibition they're having at Kensington; but I don't care for their exhibitions. I said to him, 'You go by yourself, and I'll look in at my old hospital and see if things are much changed.' And they are in some things. There's a lot more flowers about than there used to be, and there didn't used to be pianos, but it was very pleasant all the same, and everybody was as kind as kind. It was a very happy three months I spent here, and the good it did me no one can tell."

"I am glad of that; but, to tell the truth, I don't remember your face. Is it long since you were here?"

"Twenty years, sir. Twenty years last month since I came to the hospital, and nobody thought I had a year's life in me. And I don't think I would have had but for the way I was treated here. 'Going into a decline.' That was what the neighbours said, and what I thought myself. Ah, well! I've outlived many a one as looked stronger than me, and I'm hale and well to this day. And it's all due to this blessed place, and the care and kindness I got here."

"That's very good news, and we are very pleased to see you," said the secretary. "Go round and see everything you can, and tell any of the patients you like what you have told me. We'll make you an object lesson, and use you to encourage the others to think that they too may come back here as visitors some day, looking as well as you do."

### No Room! The Demand and the Supply.

"I am afraid I have kept you waiting," the secretary said, returning to the party; "but a visitor like this is a testimony to the value of our work that we prize greatly. It makes us feel thankful that, in spite of imperfections or failures, we are able to preserve and prolong many precious lives, though it leaves us the regret that we must leave an immense amount of suffering untouched. There is no more saddening sentence than that which hospital officials have to repeat so often—'no room!'"

"But I should have thought," said Mr. Curzon, "that there was enough hospital accommodation in London for all who need it."

"I won't go into the general question," said the secretary; "but with regard to our own specialty I must remind you that cases of consumption are not received into most general hospitals, and that we, in common with other hospitals for consumption, receive patients from every part of the kingdom. There are 715 beds available in the metropolitan hospitals for consumption and chest diseases, and in this computation I include the Ventnor hospital, which receives a certain amount of metropolitan support and has its offices here. Against this put the fact that in England and Wales about 39,000 people die every year of consumption. I do not say that hospital treatment could save all these; but I do say with the firmest conviction that a great number of them could be saved, and a still larger could have their lives prolonged and their passage through the dark valley made easier."

### Results.

"You set me thinking," said Mr. Curzon.

"I am to hear it. I wish I could set all the world thinking, rich and poor. We need money; we need the large subscriptions of the rich and well-to-do, and we need the little thank-offerings of the poor. And, Mr. Curzon, we need men—men who will give thought and care and advice, who will impart to this sacred work of caring for God's poor and afflicted, some of the vitality, some of the energy, they apply to their own affairs. Will you be one of those men?"

"Well, I'll think about it," said Mr. Curzon. "I must say that I never expected to be so thoroughly interested in a hospital as I have been in this."

Mr. Curzon thought to some purpose, for at the next meeting of governors the secretary announced that Mr. John Curzon had become a life governor of the hospital, having given a donation of fifty guineas. This has by no means exhausted his generosity. Two memorial beds now bear the names of his parents, and the secretary often says that no one takes a more intelligent and lively interest in the affairs of the hospital than he. And the concert organised last winter by Mrs. Curzon was one of the most successful entertainments of the season.

A letter which the secretary received only a few weeks ago, also gave him great satisfaction. The postmark was Madeira, and the contents ran thus :

"Dear Sir,

"I must recall myself to your knowledge as a former patient at the Hospital for Consumption. The treatment there and the great kindness I received from both officials and visitors did me a great deal of good, and a change to this climate has effected what I may practically regard as a cure. I have again taken up the work of my profession, and am rejoicing in a prospect of life, usefulness, and prosperity I had at one time ceased to hope for. I enclose a cheque for £5, and hope to continue the sum as an annual subscription to the hospital, to which I owe a new lease of life.—Yours faithfully,

"Charles Evans, M.B."

One word in conclusion. In this age it seems inevitable that everything should be told as a story, if it is to be generally read. But I would ask those who read this to remember that though told in narrative form, it is strictly true. The characters are invented, but there is not a single incident here related that is not strictly matter of fact. The work and wants of hospitals for consumption have not been exaggerated ; the incidents of hospital life, the words of patients have been given in their least dramatic form, and have been chosen because they are the things that happen, and the words that are uttered most frequently. We have given nothing that is *outré* or exceptional, still less have we invented a pathetic tale to touch weak minds. For those who love their kind, the simple truth, plain and unvarnished, will suffice.

